# Mutant p53 induces EZH2 expression and promotes epithelial–mesenchymal transition by disrupting p68-Drosha complex assembly and attenuating miR-26a processing

**DOI:** 10.18632/oncotarget.6350

**Published:** 2015-11-18

**Authors:** Fei-Zhou Jiang, Yin-Yan He, Hui-Hui Wang, Hui-Lin Zhang, Jian Zhang, Xiao-Fang Yan, Xiao-Jun Wang, Qi Che, Jie-Qi Ke, Zheng Chen, Huan Tong, Yong-Li Zhang, Fang-Yuan Wang, Yi-Ran Li, Xiao-Ping Wan

**Affiliations:** ^1^ Department of Obstetrics and Gynecology, Shanghai General Hospital, Shanghai Jiao Tong University School of Medicine, Shanghai, China; ^2^ Department of Obstetrics and Gynecology, Shanghai Jiaotong University Affiliated International Peace Maternity & Child Health Hospital of the China Welfare Institute, Shanghai, China; ^3^ Department of Obstetrics and Gynecology, Shanghai First Maternity and Infant Hospital, Tongji University School of Medicine, Shanghai, China

**Keywords:** endometrial carcinoma, p53, EZH2, p68, miR-26a, EMT

## Abstract

The tumor suppressor p53 and the transcriptional repressor Enhancer of Zeste Homolog 2 (EZH2) have both been implicated in the regulation of epithelial-mesenchymal transition (EMT) and tumor metastasis via their impacts on microRNA expression. Here, we report that mutant p53 (mutp53) promotes EMT in endometrial carcinoma (EC) by disrupting p68-Drosha complex assembly. Overexpression of mutp53 has the opposite effect of wild-type p53 (WTp53), repressing miR-26a expression by reducing pri-miR-26a-1 processing in p53-null EC cells. Re-expression of miR-26a in mutp53 EC cells decreases cell invasion and promotes mesenchymal-epithelial transition (MET). Rescuing miR-26a expression also inhibits EZH2, N-cadherin, Vimentin, and Snail expression and induces E-cadherin expression both *in vitro* and *in vivo*. Moreover, patients with higher serum miR-26a levels have a better survival rate. These results suggest that p53 gain-of-function mutations accelerate EC tumor progression and metastasis by interfering with Drosha and p68 binding and pri-miR-26a-1 processing, resulting in reduced miR-26a expression and EZH2 overexpression.

## INTRODUCTION

Endometrial carcinoma (EC) is a common gynecologic malignancy classified into two subtypes, type I and type II [1]. Type I EC occurs in ~85% of patients and is often estrogen receptor positive with well-differentiated tumors of low grade and good prognosis. Type II EC is a biologically progressive group of ECs that includes papillary serous carcinoma and clear cell carcinoma [2] and accounts for the remaining 15% of EC cases but is responsible for a disproportionate number of relapses. Patients with type II EC tumors have a 5-year survival rate of only 44% [3]. Molecular mechanisms explaining the development and progression of type II EC are still unknown.

The tumor suppressor p53 is a transcription factor that inhibits malignant transformation by inducing cell cycle arrest, senescence, and apoptosis [4–6]. p53 is mutated in 50% of human cancers. The mutant p53 (mutp53) may not only lose its tumor-suppressor functions, it may also acquire oncogenic gain of function (GOF) [7–9]. Integrated genomic characterization of EC tumors revealed that p53 missense mutations were frequent in type II EC, suggesting a role for mutp53 in EC progression [10].

Enhancer of Zeste Homolog 2 (EZH2) is the catalytic subunit of the Polycomb Repressive Complex 2 (PRC2), which silences gene transcription through trimethylation of histone H3 on lysine27 (H3K27me3) [11–13]. EZH2 is often overexpressed in prostate and breast cancers, where it promotes tumor formation and progression and correlates with a poor prognosis [14–16]. EC tumors overexpress EZH2, with higher expression in type II than type I EC (63% *vs* 7.6%) [17], Aberrant p53 status is also associated with EZH2 overexpression [18]; however, the mechanistic links between mutp53 and EZH2 are unknown.

Here, we confirm previous results and identify a novel molecular mechanism by which mutp53 promotes EC invasion and epithelial-mesenchymal transition (EMT). We show that mutp53 interferes with associations between the Drosha complex and p68, leading to attenuation of pri-miR-26a-1 processing and overexpression of its downstream target, EZH2.

## RESULTS

### EZH2 and mutp53 are highly expressed in EC specimens

**Figure 1:**
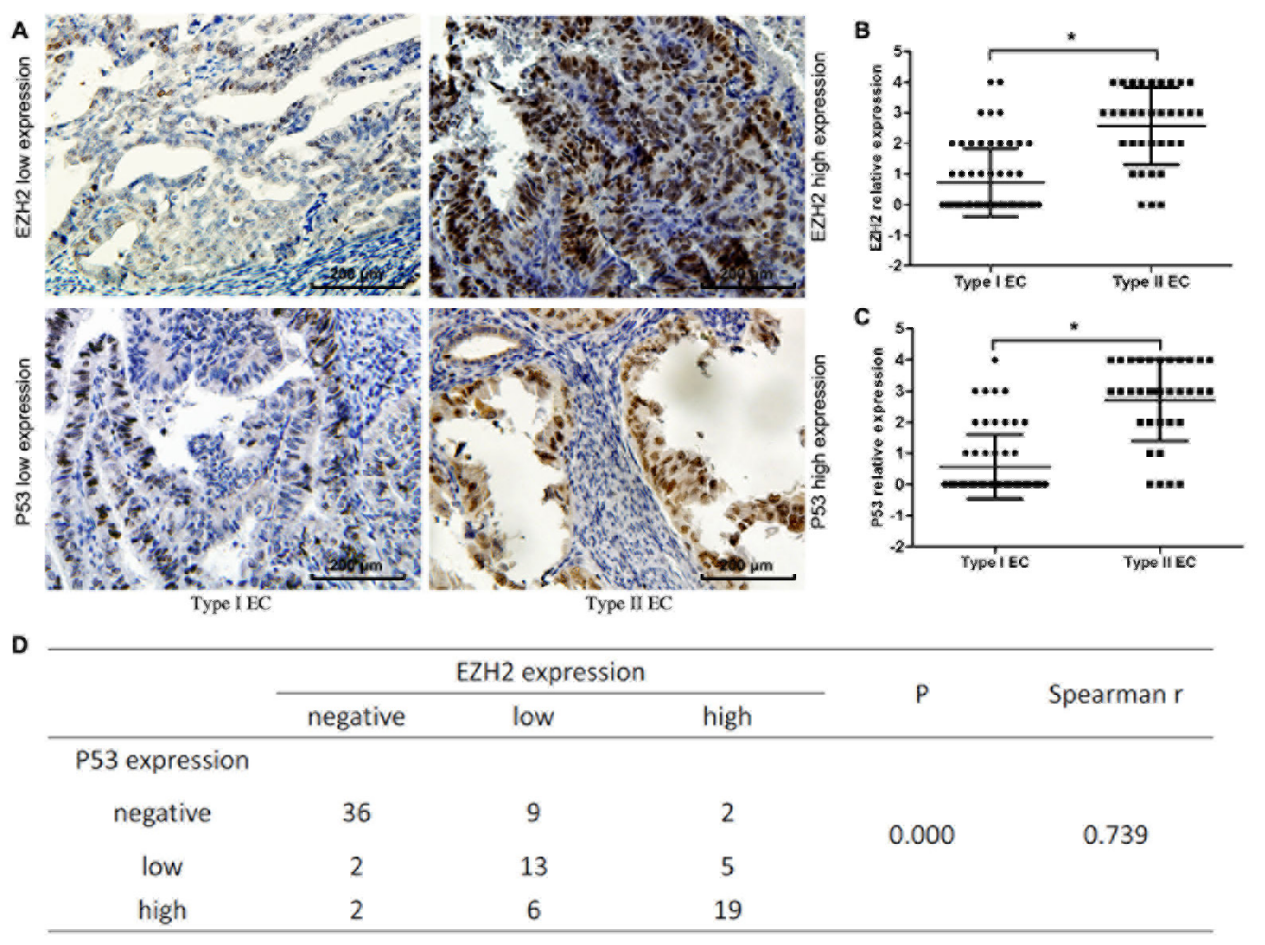
Mutp53 and EZH2 expression in EC tissues **A.** Representative expression of mutp53 and EZH2 in type I EC and type II EC tissues with mutp53 and EZH2 high expression (right panel) and low expresssion (left panel). **B.**~**C.** Type II EC samples had increased mutp53 and EZH2 expression when compared with type I samples (*P < 0.001). **D.** EZH2 expression was positively correlated with mutp53 expression in ECs (Spearman correlation r = 0.739).

We examined 124 endometrium specimens: 24.2% were identified as normal, whereas 48.4 % were type I EC, and 27.4% were type II EC. The mean age was 51.5 years (range, 26-80 years) in the normal endometrium group, 50.8 years (range, 28-72 years) in the type I EC group, and 54.8 years (range, 40-82 years) in the type II EC group. A good correlation (75-100%) between positive immunohistochemistry and p53 mutations was observed in mammary and EC [19]. Of 30 normal endometrial specimens stained by immunohistochemistry (IHC), only one expressed EZH2 at low levels and none expressed p53. In EC tissues, only 10% of type I tumors had high EZH2 expression whereas 58.8% of type II tumors expressed high levels of EZH2 (Table 1, *P* < 0.001; Figure 1A&1B, *P* < 0.001). Consistent with its role as an epigenetic modifier and transcriptional regulator, EZH2 expression was predominantly localized in the nuclei of EC cells. Mutp53 shared the same pattern of expression across tissues as EZH2 (6.7% in type I *versus* 67.6% in type II tumors, Figure 1A & 1C, *P* < 0.001). Moreover, EZH2 expression was positively correlated with mutp53 expression in type II EC (Spearman correlation r = 0.739, Figure 1D). As shown in Table 1, EZH2 expression was higher in cases with a high FIGO stage (*P* < 0.001), a deep myometrial invasion (*P* = 0.023), a high histological grade (*P* < 0.001), and lymph node metastasis (*P* = 0.002). These data suggest a common mechanism underlying EZH2 and mutp53 expression in EC tissues.

**Table 1:** Association analyses between the expression of EZH2 and clinicopathologic factors of endometrial carcinoma

Variables	Case no.	EZH2 expression			χ2	*P*- value
		Negative	Low	High		
Age (year)						
<55	61	28	19	14	1.959	0.376
≥55	33	12	9	12		
Histological type						
Type I	60	37	17	6	33.062	<0.001
Type II	34	3	11	20		
Histological grade						
G1	58	34	17	7	26.518	<0.001
G2	22	5	8	9		
G3	14	1	3	10		
FIGO stage						
I-II	67	35	22	10	19.545	<0.001
III-IV	27	5	6	16		
Lymph node metastasis						
NO	73	36	21	16	12.334	0.002
Yes	21	4	7	10		
Depth of endometrial invasion						
≤50%	73	36	21	16	7.520	0.023
>50%	21	4	7	10		
p53 expression						
Negative	47	36	9	2	63.414	<0.001
Low	20	2	13	5		
High	27	2	6	19		

### Mutp53 induces EZH2 expression and inhibits miR-26a expression

**Figure 2:**
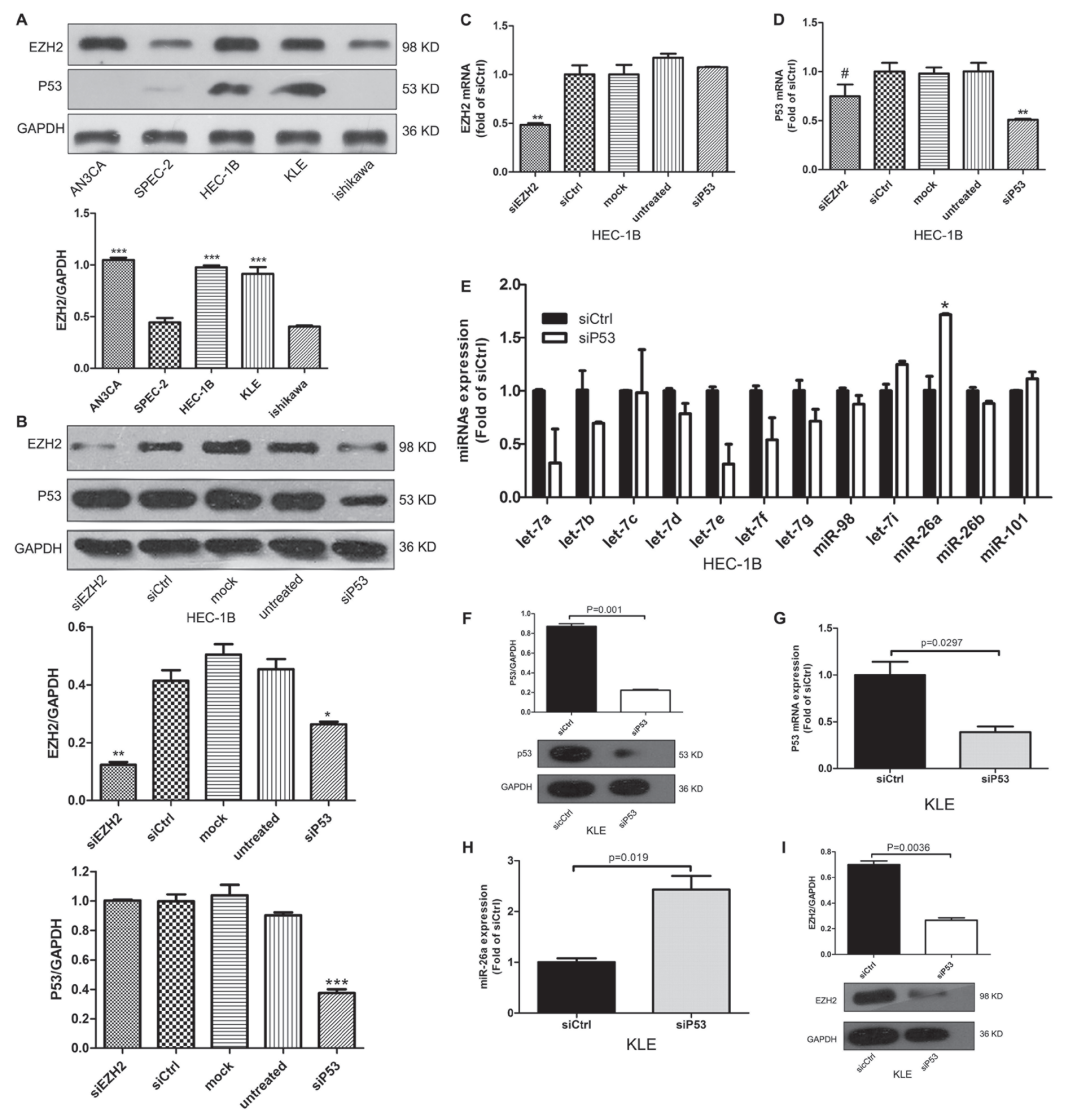
Mutp53 induces EZH2 expression and inhibits miR-26a expression **A.** EZH2 expression was examined in five EC cell lines including HEC-1B (p53-R248Q mutation) and KLE (p53-R175H mutation) by western blot (***P < 0.001). **B.** Mutp53 and EZH2 protein expression after siRNA knockdown of EZH2 or mutp53 (*P < 0.05, **P < 0.01, ***P < 0.001). **C.** *EZH2* mRNA expression was not altered 72 h after siRNA knockdown of mutp53 (**P < 0.01). **D.** Mutp53 mRNA levels decreased slightly after siRNA knockdown of EZH2 (# > 0.05, **P < 0.01). **E.** miR-26a expression was increased after siRNA knockdown of mutp53 (*P < 0.05). **F.**~**G.** Mutp53 protein and mRNA expression at 72 h after transfection of siRNA in mutp53 KLE (R175H) cells. **H.**~**I**. Knockdown of mutp53 induced miR-26a expression and suppressed EZH2 protein expression in KLE cells.

We next examined the expression of EZH2 and p53 in five EC cell lines, including two lines with mutp53: HEC-1B (p53-R248Q) and KLE (p53-R175H). EZH2 was overexpressed in HEC-1B, KLE, and AN3CA cells compared with SPEC-2 and Ishikawa cells (Figure 2A). To investigate the relationship between EZH2 and mutp53, we used siRNA to knockdown mutp53 expression in HEC-1B cells. EZH2 protein expression was decreased with mutp53 knockdown, but EZH2 mRNA expression remained unchanged (Figure 2B & 2C). In contrast, siRNA knockdown of EZH2 had no effect on p53 protein expression, but slightly reduced its mRNA expression (Figure 2B & 2D, *P* > 0.05). Therefore, we hypothesized that mutp53 induces EZH2 overexpression through inhibition of a miRNA.

To determine which miRNAs are regulated by mutp53, we used qRT-PCR and the biological predicition software, TargetScan. Down-regulation of mutp53 by siRNA increased miR-26a expression in HEC-1B (Figure 2E) and KLE (Figure 2F, 2G, 2H & 2I) cells, suggesting that endogenous mutp53 represses miR-26a expression.

### Mutp53 attenuates pri-miR-26a-1 processing and disrupts p68-drosha complex assembly

**Figure 3:**
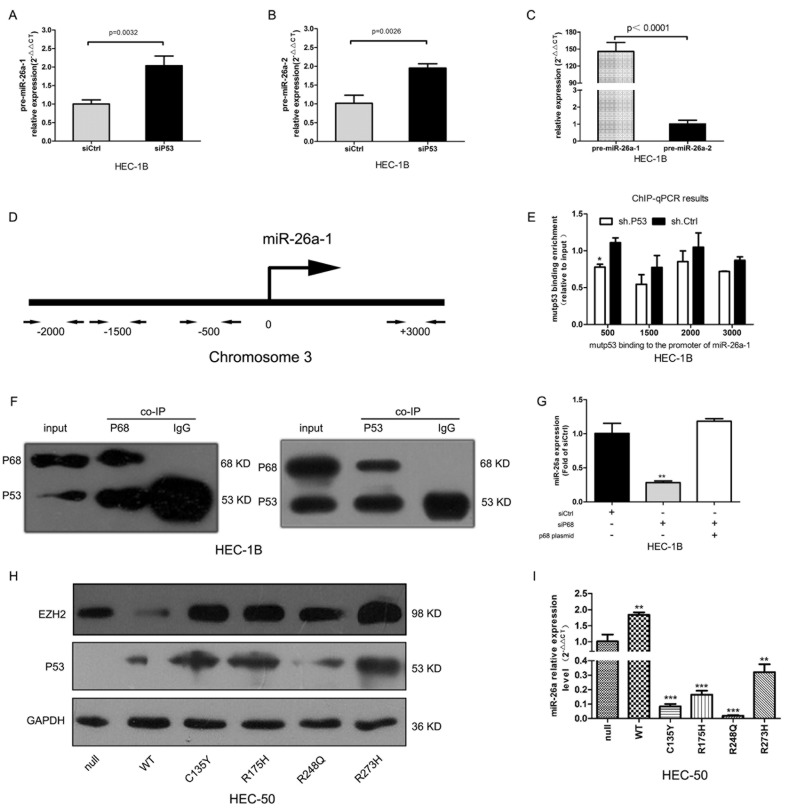
Mutp53 inhibits miR-26a via interactions with p68 and enhances EZH2 expression **A.**~**B.** pre-miR-26a-1 and pre-miR-26a-2 expression increased nearly 2-fold in the sip53 group compared with controls. **C.** pre-miR-26a-1 is expressed nearly 150-fold more than pre-miR-26a-2 in HEC-1B cells after silencing mutp53. **D.** Schematic of the miR-26a-1 regulatory region. **E.** A summary of ChIP assay results for mutp53 binding in HEC-1B cells transfected with sh.p53 or sh.Ctrl expression plasmid (*P < 0.05). **F.** Reciprocal co-IP (co-immuniprecipitation) of p53 and p68. p68 was immunoprecipitated using the p68-specific antibody and p68 and p53 expression detected by Western blot (left panel). p53 was immunoprecipitated using a p53-specific antibody and p53 and p68 expression detected by Western blot (right panel). **G.** miR-26a expression decreased after p68 knockdown and was rescued by re-expression of p68 (**P = 0.004). **H.** p53 and EZH2 protein expression in p53-null HEC-50 cells transfected with different mutp53 expression plasmids. **I.** miR-26a expression was induced by WTp53 and suppressed by mutp53 (**P < 0.01, ***P < 0.001).

miR-26a is encoded by the two genes, miR-26a-1 and miR-26a-2, which are located on chromosomes 3 and 14, respectively [20]. To elucidate which gene is upregulated after mutp53 inhibition, we examined the expression of miR-26a precursors. After knockdown of mutp53 both pre-miR-26a-1 and pre-miR-26a-2 were increased about 2-fold over the control group (Figure 3A & 3B, *P* < 0.01). However, the baseline expression of pre-miR-26a-1 was nearly 150-fold that of pre-miR-26a-2 in HEC-1B cells (Figure 3C, *P* < 0.0001). Therefore, the increase in miR-26a is likely due to miR-26a-1 overexpression.

Wild-type p53 (WTp53) activation by genotoxic stress directly induces miR-26a expression *via* binding of the −500, −1500, and −2000 regions of the miR-26a-1 gene [20]. To determine whether mutp53 is able to bind the miR-26a-1 gene promoter, we performed a ChIP assay using a p53-specific antibody and a series of primers spanning the upstream region of the gene (Figure 3D). Maximal binding of mutp53 was detected at the −500 region with only a 1.4-fold decrease in sh.p53 (shp53 plasmid carrying small hairpin RNA targeting p53, sh.p53) treated cells compared with controls (Figure 3E, *P* < 0.05). However, WTp53 bound all three upstream regions in both p53-null HEC-50 cells transfected with WTp53 and in wi-38 cells with endogenous WTp53 expression (supplementary figure 1). These results suggest that mutp53 binding to the miR-26a-1 gene promoter is impaired and that mutp53 might suppress miR-26a expression by a gain-of-function.

p68 interacts with WTp53, suggesting that it may form a multiprotein transcription regulatory complex [21]. Therefore, we used co-immunoprecipitation experiments to investigate whether p68 interacts with mutp53. As shown in Figure 3F, p68 reciprocally binds mutp53 in HEC-1B cells. Knockdown of p68 decreased miR-26a expression, while re-expression of p68 rescued miR-26a expression (Figure 3G, *P* = 0.004). Similar results were found in experiments with p53-null HEC-50 EC cells (supplementary figure 2A). To study the effects of several tumor-derived transcriptionally inactive p53 mutants (C135Y, R175H, R248Q, and R273H) on miR-26a expression, we transfected different mutp53 expression plasmids into p53-null HEC-50 cells. While WTp53 induced miR-26a expression, mutp53 suppressed its expression (Figure 3H & 3I). Conversely, EZH2 expression was downregulated by WTp53 and upregulated by mutp53 (Figure 3H).

**Figure 4:**
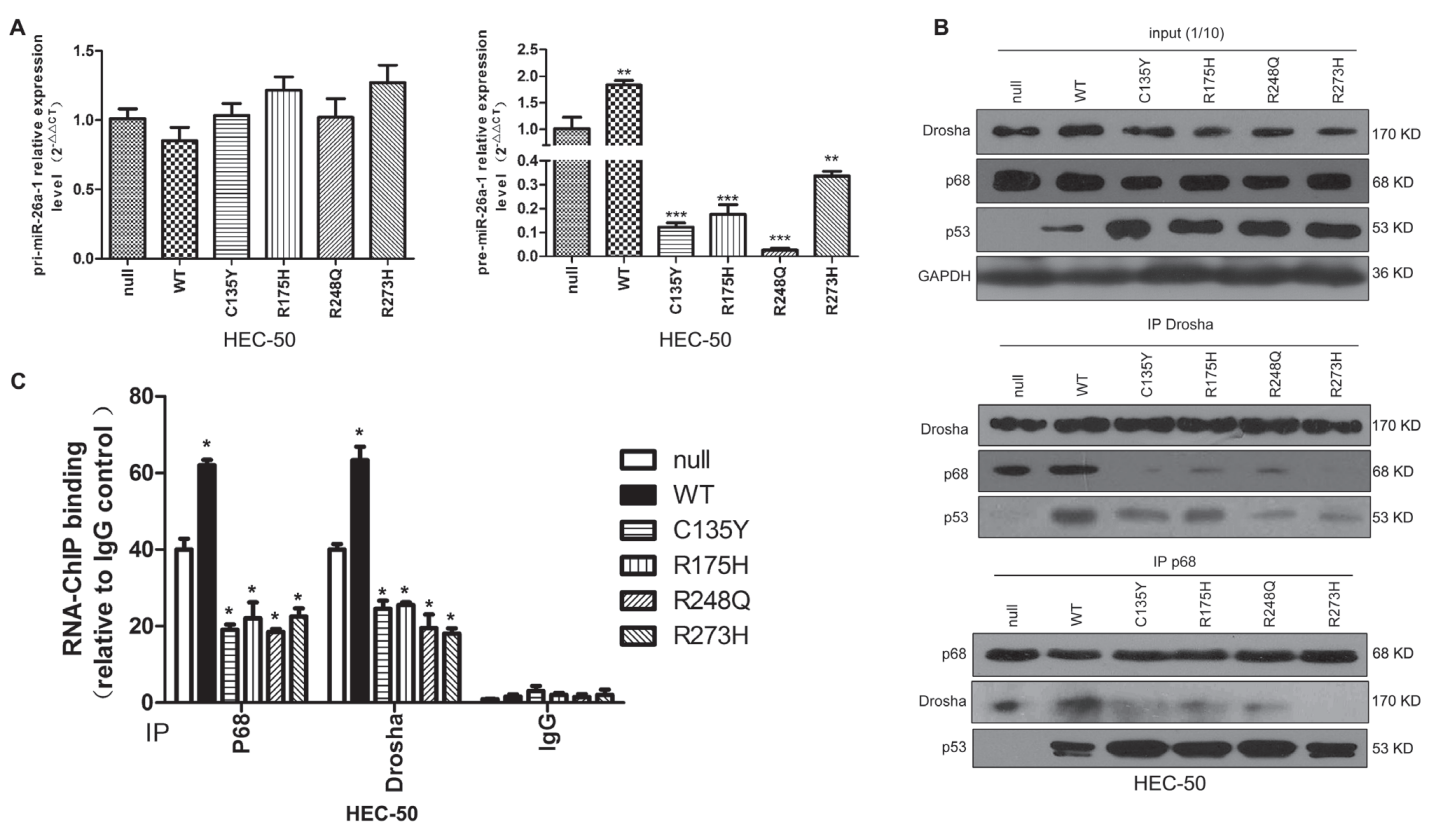
miRNA processing is deregulated by mutp53 **A.** p53-null HEC-50 cells were transfected with different p53 expression plasmids. The amount of pri-miR-26a-1 and pre-miR-26a-1 were examined (**P < 0.01 *vs* null, ***P < 0.001 *vs* null). **B.** Immunoprecipitation (IP) assays were performed after transfection with different p53 expression plasmids. **C.** RNA-ChIP analysis. After transfection of p53-null HEC-50 cells with different p53 expression plasmids, endogenous proteins were immunoprecipitated by anti-p68 or anti-Drosha antibodies and subjected to RT–PCR analysis with miR-26a-1 (*P < 0.05 *vs* null).

In miRNA biogenesis, the pri-miRNAs are cleaved into pre-miRNAs by the nuclear RNase III, Drosha, and further processed to mature miRNAs by cytosolic Dicer. The Drosha complex comprises Drosha and multiple RNA-associated proteins, including p68 [22], which is required for the maturation of some, but not all miRNAs [23]. We therefore examined the effects of tumor-derived p53 mutants on miRNA processing by transfecting them into p53-null HEC-50 cells. p53 mutant cells had reduced pre-miR-26a-1 expression but no change in pri-miR-26a-1 expression. In contrast, WTp53 transfection increased pre-miR-26a-1 expression (Figure 4A). We next examined the effects of mutp53 expression on Drosha complex formation. Cells transfected with mutp53 had decreased interactions between Drosha and p68, while cells with WTp53 showed a modest increase in the Drosha-p68 association (Figure 4B). RNA-ChIP analysis revealed that cells with mutp53 had decreased associations between pri-miR-26a-1 and p68/Drosha (Figure 4C). These results suggest that tumor-derived p53 mutations might confer some different and opposite functions to the protein, leading to interference with miR-26a biogenesis by interference with Drosha/p68 complex assembly.

### EZH2 is the direct molecular target of miR-26a

**Figure 5:**
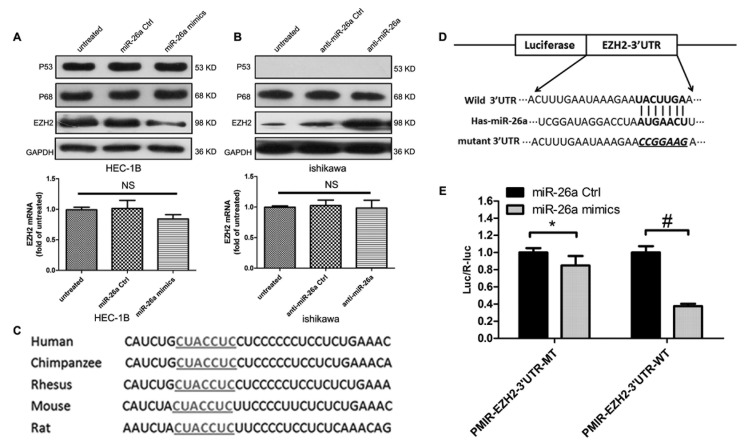
miR-26a negatively regulates EZH2 protein expression in EC cells **A.**~**B.** EZH2 protein and mRNA levels were measured in EC cells 72 h post-transfection by western blot assays (NS, not significant). **C.** Putative binding site of miR-26a on the *EZH2* 3′UTR in different species. The seed sequence is underlined. **D.** Sketch of the construction of pMIR-EZH2-3′UTR-WT or pMIR-EZH2-3′UTR-MT vectors. The mutant binding site is underlined and italicized. **E.** miR-26a mimics down-regulate luciferase activity controlled by wild-type EZH2 3′UTR (*P = 0.0122; #*P* < 0.001).

To further establish the effects of miR-26a on EZH2 expression, we examined HEC-1B cells transfected with miR-26a mimics and Ishikawa cells transfected with anti-miR-26a. Compared with controls, transfection of miR-26a mimics decreased EZH2 protein expression (Figure 5A). In contrast, EZH2 protein expression was increased in Ishikawa cells transfected with anti-miR-26a (Figure 5B). Neither treatment altered EZH2 mRNA expression (Figure 5A & 5B).

TargetScan analysis indicated that the *EZH2* gene contains a highly conserved miR-26a binding site on its 3′UTR (Figure 5C). To determine whether this putative miR-26a binding site was actually regulated by miR-26a, we constructed vectors containing the wild-type or mutant 3′UTR of *EZH2* fused with the firefly luciferase gene (Figure 5D). Vectors were cotransfected into NIH-3T3 cells with miR-26a mimics or miR-26a control. Transfection efficiency was normalized by cotransfection with a Renilla reporter vector. As shown in Figure 5E, miR-26a decreased the relative luciferase activity of the wild-type *EZH2* 3′UTR vector by more than 65%, whereas the reduction in luciferase activity of the mutant *EZH2* 3′UTR vector was not as dramatic. These results suggest that miR-26a can bind to the 3′UTR of *EZH2*, and that *EZH2* may be a downstream target of miR-26a in EC cells.

### miR-26a overexpression inhibits cell proliferation and invasion

**Figure 6:**
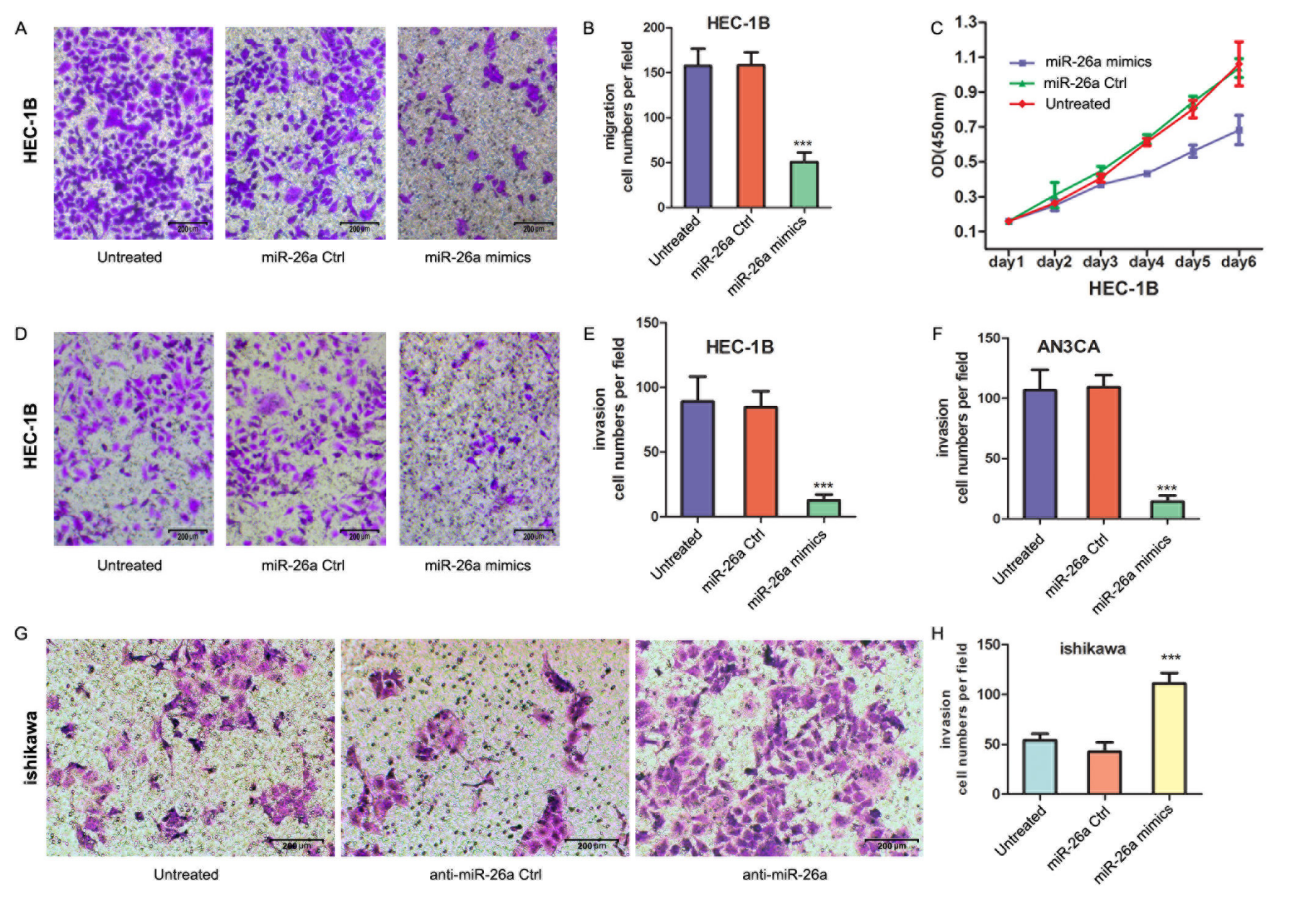
miR-26a impairs the EC cell migration and invasion **A.** HEC-1B cells were transfected with miR-26a mimics, control or none for 48 h and seeded in a transwell filter. Migrated cells on the lower surface of the transwell filter were stained and counted after 24 h. **B.** Quantification of A. Bars show mean ± SD. ***P = 0.0002. **C.** CCK8 analysis of the growth of HEC-1B cells transfected with miR-26a mimics, control or none. **D.** HEC-1B cells after transfection seeded in a transwell filter paved with marigel matrix gel. Invasive cells on the lower surface of the transwell filter were stained and counted after 24 h. **E.** Quantification of D. Bars show mean ± SD. ***P = 0.0007. **F.** The numbers of invasive cells of AN3CA. Bars show mean ± SD. ***P < 0.0001. **G.** the invasion of Ishikawa after transfection was assayed in the same way. **H.** Quantification of **G**. Bars show mean ± SD. ***P = 0.0002.

Mutp53 promotes cell migration, invasion and metastasis [24–29]. We found that stable knockdown of mutp53 in HEC-1B cell decreased invasion and colony formaton (supplementary figure 3). When miR-26a was re-expressed in p53 mutant HEC-1B cells with high EZH2 expression, it impaired the migration (Figure 6A & B, ***P = 0.0002). and proliferation (Figure 6C) of HEC-1B cells. Importantly, the effects on proliferation were observed after 3 days of transfection and not at earlier time points when the migration assays were performed, indicating the reduction in migration was not due to reduced cell numbers. miR-26a overexpression also inhibited the invasion of HEC-1B cells (Figure 6D & 6E, ***P = 0.0007) as well as invasion of poorly differentiated AN3CA cells with high EZH2 expression (Figure 6F, ***P < 0.0001). However, knockdown of miR-26a promoted Ishikawa cell invasion (Figure 6G & 6H, ***P = 0.0002).

### miR-26a promotes mesenchymal-epithelial transition (MET) of EC cells

**Figure 7:**
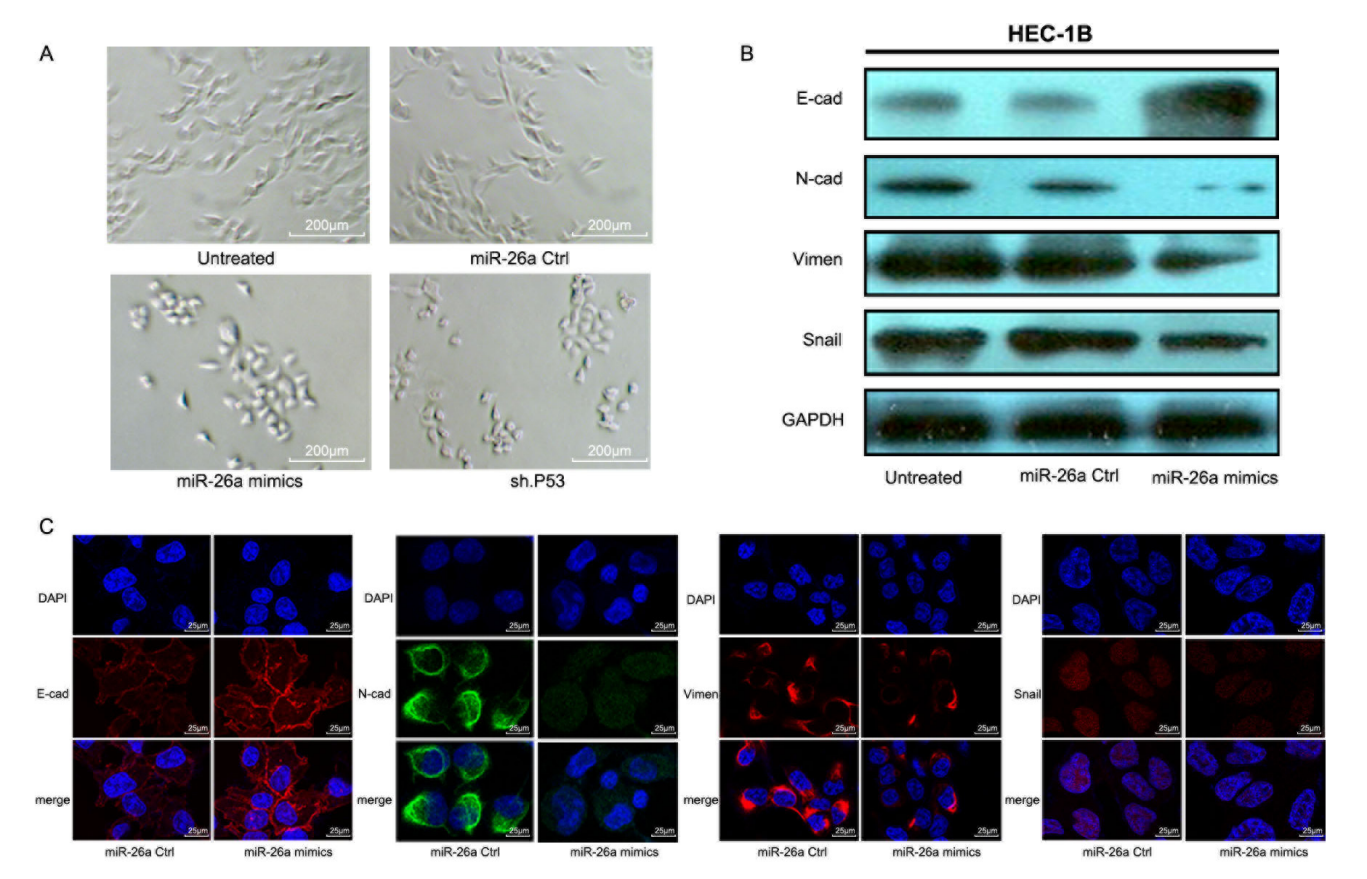
miR-26a promotes MET in HEC-1B cells **A.** Morphological changes in HEC-1B cells after different treatments. **B.** EMT-related markers were examined in HEC-1B cells transfected with miR-26a mimics, miR-26a Ctrl mimics, or none. **C.** Changes in EMT markers were analyzed using scanning laser confocal microscopy after transfection with miR-26a mimics or control.

To determine whether the miR-26a inhibition of cell invasion was due to induction of MET, we examined morphologIcal changes in HEC-1B cells transfected with miR-26a mimics. Overexpression of miR-26a induced a shift from a mesenchymal phenotype to a “paved stone” epithelial appearance, similar to changes seen in HEC-1B cells with stable knockdown of mutp53 (Figure 7A). These morphological changes were accompanied by the downregulation of mesenchymal genes, including N-cadherin, Snail, and Vimentin, and increased expression of the epithelial marker E-cadherin (Figure 7B). When examined in detail with scanning laser confocal microscopy, exogenous miR-26a expression induced E-cadherin expression around cell-to-cell contacts and reduced Vimentin and N-cadherin expression in the cytoplasm, while Snail expression was reduced in the nuclei (Figure 7C).

### miR-26a acts as a tumor suppressor in a mouse tumor xenograft model

**Figure 8:**
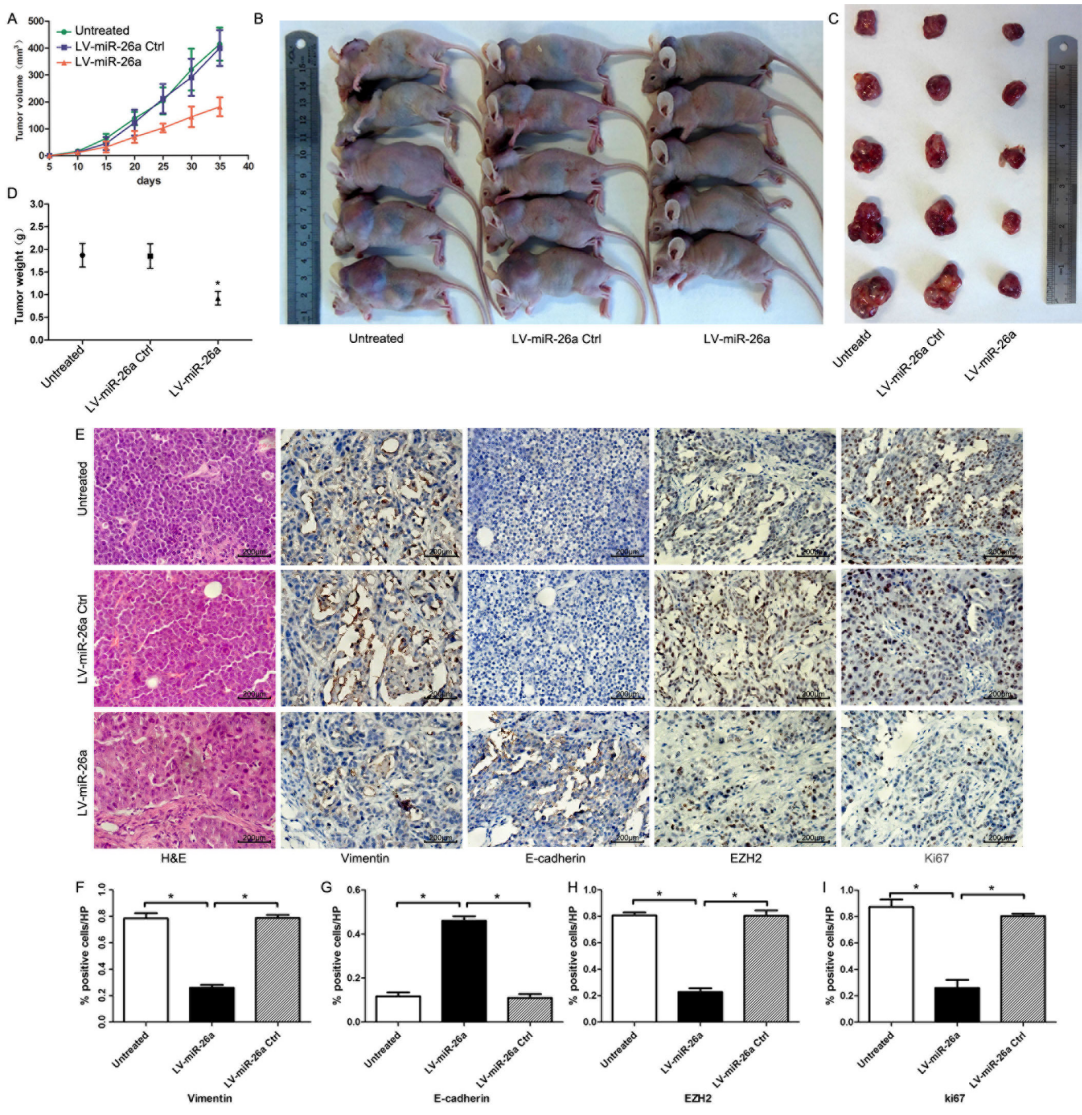
Tumorigenicity assay in nude mice **A.** Tumor growth curve in nude mice. After tumor cells were injected subcutaneously into the neck of nude mice, the short and long diameters of the tumors were measured every 5 days and tumor volumes (mm3) were calculated. **B.** The nude mice with tumor formations. **C.** Photograph of tumors derived from LV-miR-26a, LV-miR-26a Ctrl or untransfected HEC-1B cells in nude mice. **D.** Weights of tumors. *P < 0.05 as compared with either untreated group or LV-miR-26a Ctrl group. **E.** Representative HE staining histopathologic image of tumor tissues in mice (left panel). Vimentin, E-cadherin, EZH2, and Ki67 expression of tumors were detected by immunohistochemical technique (right panel). **F.**~**I.** Quantification of Vimentin, E-cadherin, EZH2, and Ki67 expression in E. *P < 0.01.

To further determine the role of miR-26a in the regulation of MET, we performed tumor xenograft experiments using HEC-1B cells. Subcutaneous tumor formation was observed in all nude mice 10 days after injection. During the 35 day follow-up period, the tumor volumes increased (Figure 8A). However, at 35 days, the size and weights of tumors were smaller in animals treated with LV-miR-26a than those that were untreated or treated with a LV-miR-26a control (Figure 8B, 8C & 8D). Post-mortem, tumor tissues were embedded in paraffin and stained with hematoxylin and eosin (H&E) for histological examination (Figure 8E, left panel). Immunohistochemical staining of Vimentin and E-cadherin revealed lower Vimentin expression and higher E-cadherin expression in LV-miR-26a tumors than in untreated or mock controls (Figure 8E, 8F & 8G). Our *in vitro* studies suggested that *EZH2* is the direct downstream target of miR-26a; therefore, we examined EZH2 protein expression in tumor tissues using IHC. EZH2 expression was reduced in the LV-miR-26a group compared to controls (Figure 8E & 8H), leading further support to the hypothesis that *EZH2* is regulated by miR-26a. In addition, the LV-miR-26a group had lower proliferation indices than the controls (Figure 8E & 8I).

### Plasma miR- 26a expression may be a diagnostic tool for EC patients

**Figure 9:**
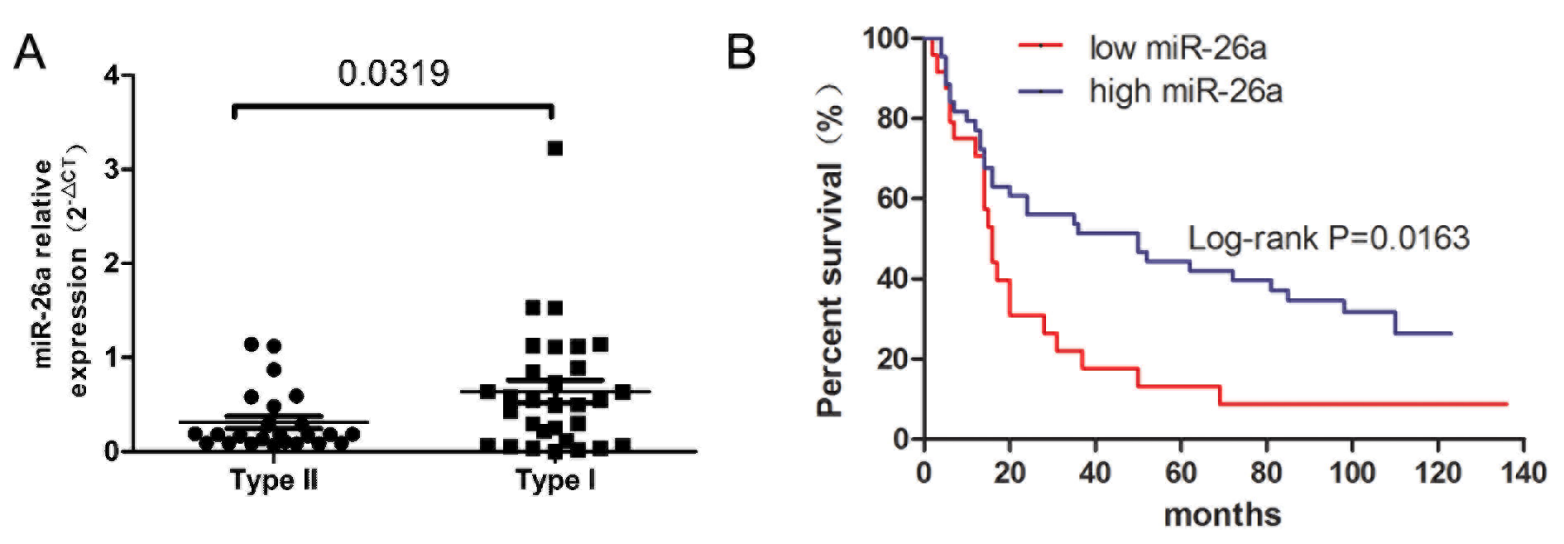
miR-26a expression and prognosis of EC patients **A.** miR-26a expression was reduced in type II EC patient serum compared to type I. **B.** Kaplan–Meier overall survival curve according to miR-26a expression in EC patient serum (Log-rank *P* = 0.0136).

Expression of miR-26a was significantly reduced in type II EC serum compared to type I EC (Figure 9A). Moreover, patients with high miR-26a expression survived longer than patients with lower expression (Figure 9B).

## DISCUSSION

Even when detected at an early-stage, most type II ECs already have distant metastasis. However, the mechanism underlying metastasis is still unknown. The importance of p53 in preventing tumor formation is indicated by high rates of mutations in the p53 pathway in nearly all cancer types [30]. Integrated genomic characterization of EC indicates that recurrent p53 mutations are common in type II EC (90%) [10], suggesting mutp53 promotes EC progression. Mice with endometrium-specific deletion of p53 exhibit histological changes that are identical to known precursor lesions for type II EC in humans and develop carcinomas exhibiting features of all type II subtypes [6].

It has been reported that WTp53 binds directly to the EZH2 promoter and represses EZH2 expression, leading to cell senescence [31]. These results differ from our finding that WTp53 inhibits EZH2 expression indirectly *via* miR-26a at the posttranscriptional level. Others have found that mutp53, but not WTp53, is reduced in response to EZH2 knockdown [32]. However, DZNep (3-deazaneplanocin A) stabilizes WTp53 by reducing ubiquitin conjugation through USP10 upregulation, resulting in p53 accumulation and activation of its downstream target genes [33]. Consequently, WTp53 cancer cells are sensitive to DZNep treatment. Thus, the relationship between EZH2 and p53 may be complex and cell-type dependent.

Most somatic mutations (82%) in p53 are single nucleotide missense substitutions located in the DNA binding doman (DBD), which encompasses p53 exons 5-8. Mutations in codons 248 and 273 are classified as DNA contact mutations, while alterations in codon 175 are considered conformational mutations [34]. However, no matter how these hot-spot mutants are classified, all have impaired sequence-specific DNA-binding capacity resulting in a loss of transcriptional activity [35–37]. Recent studies have shown that WTp53 induces miR-26a expression by directly binding to the −500, −1500, and −2000 regions of *miR-26a-1* [20]. We failed to find mutp53 binding in these upstream regions suggesting that mutp53 does not suppress miR-26a expression *via* a dominant negative mechanism. Consistent with that finding, we also demonstrated that mutp53 has reduced DNA-binding ability. One limitation of our study is that we chose to examine only hot-spot p53 mutations; there may be other p53 mutations that have different effects.

We demonstrated that p53 mutations can induce EMT and increase the invasiveness of EC cells by regulating a large set of EMT-associated genes. More importantly, we provide an underlying mechanism for mutp53-enhanced metastasis: p53 mutations not only impair its ability to bind the promoter of miR-26a-1, but also impair p68-Drosha complex assembly and attenuate miR-26a-1 processing. Together, this results in a reduction in miR-26a expression and upregulation of EZH2 expression. Our results suggest that p53 mutations result in “gain-of-function” oncogenic properties that can contribute to cell proliferation, survival, and metastasis. Notably, the effects of mutp53 on EMT and cell invasion can be partially abolished by restoration of miR-26a expression. Re-expression of miR-26a may therefore inhibit tumor metastasis and progression, providing a potential therapeutic use in patients with EC.

mutp53 drives EMT *via* various signaling pathways, including the miR-130b-ZEB1 axis and Rab coupling protein (RCP)-dependent receptor recycling [9, 27]. At the molecular level, EMT occurs as a result of the activity of several transcription factors, including Twist, Snail, and ZEB1/2, which repress expression of the epithelial marker E-cadherin and promote expression of the mesenchymal markers N-cadherin and Vimentin. DAB2IP, which is negatively regulated by EZH2, is an important suppressor of EMT [38]. However, EMT initiation and regulation is still not fully understood.

We previously showed that DICER1, a key enzyme catalyzing miRNA biosynthesis, is expressed at lower levels in AN3CA than RL95-2, Ishikawa, or KLE cells (supplementary figure 2B) [39, 40]. We also showed that levels of Let-7b expression are lower in AN3CA cells than in RL95-2, Ishikawa, or KLE cells (supplementary figure 2C). Two recent studies showed that overexpression of Let-7b reduces EZH2 protein levels [41, 42]. These results may explain why AN3CA cells express high levels of EZH2 with undetectable p53 expression, but this requires further investigation.

In summary, we identified a mechanism by which mutp53 exerts oncogenic effects and promotes EMT in EC by disrupting p68-Drosha complex assembly and decreasing miR-26a production. This results in increased EZH2 expression and promotes EC tumor progression. Our results provide further evidence of the vital roles played by miRNAs in EC tumorigenesis. Although miRNA-based therapeutics are still in their infancy, our findings suggest that miR-26a could be a potential target in the treatment of EC [43].

## MATERIALS AND METHODS

### Cell culture

The EC cell lines KLE, HEC-1B, and AN3CA were obtained from American Type Culture Collection (ATCC, Manassas, Va) and Ishikawa, SPEC-2 and p53-null HEC-50 were obtain from the Cell Bank of the Chinese Academy of Sciences (Shanghai, China). All cell lines were cultured in DMEM/F12 medium containing 10% fetal bovine serum.

### Patients and samples

Tissue samples for IHC and serum were obtained from 94 patients with EC and 30 patients with normal endometrium who underwent surgical resection at Department of Obstetrics and Gynecology, Shanghai General Hospital from 1997 to 2012, and each case had up to 15 years of clinical follow-up information. The project was approved by the Institutional Review Board of Shanghai General Hospital, and informed consent was obtained from all patients before the study.

### Reagents

Synthetic, chemically modified short single- or double-stranded RNA oligonucleotides: miR-26a mimics, miR-26a control (Ctrl), anti-miR-26a, anti-miR-26a Ctrl, p68 siRNA (sip68), p53 siRNA (sip53), and EZH2 siRNA (siEZH2) were synthesized from Shanghai GenePharma Co., Ltd. Commercial miR-26a expression Lentivirus and counterpart control vector were purchased from Shanghai GenePharma Co., Ltd. Commercial p68 expression plasmid and sh.p53 plasmid carrying small hairpin RNA targeting p53 were purchased from Shanghai Gene-Chem Co., Ltd. Commercial mutp53 expression plasmids and wild-type p53 expression plasmid were constructed and provided by GuangZhou Biosicen Biotechnology CO., Ltd. All oligonucleotide sequences are listed in supplementary table 1.

### Immunohistochemistry (IHC)

Antibodies used for IHC were: EZH2 (5246; 1:50; CST), p53 (2527; 1:200; CST), Vimentin (5471; 1:100; CST), E-cadherin (3195; 1:400; CST), and Ki67 (BM2889; 1:200; Boster). For evaluation of EZH2 and p53 IHC staining, the staining intensity was scored as 0 (negative), 1 (weak), 2 (strong). The extent of staining was scored as 0 (< 25%), 1 (25~75%), and 2 (> 75%). The final immunoreactivity scores were obtained by adding these two scores. Tissues having a final immunoreactivity score of 3~4 and 1~2 were considered to be high expression and low expression, respectively. Staining was scored independently by two pathologists without knowledge of the clinicopathological findings.

### RNA isolation and qRT- PCR

Briefly, total RNA was extracted from cells and serum using TRIzol^®^ and TRIzol^®^ LS Reagent (Life Technologies, USA) following the supplier's instructions. All primers and reagents for mature miRNA analysis were purchased from Life Technologies according to manufacturer's instructions. U6 snRNA served as an endogenous control for normalization. Other specific primers for qRT- PCR are listed in supplementary table 2.

### Western blot analysis, ChIP and RNA-ChIP

Total cell lysis and western blot analysis were performed as described previously using the following antibodies [44]: EZH2 (5246; 1:1000; CST), p53 (2527; 1:1000; CST), N-cadherin (13116; 1:1000; CST), E-cadherin (3195; 1:1000; CST), Vimentin (5741; 1:1000; CST), Snail (3879; 1:1000; CST) and GAPDH (5632-1; 1:2000; Epitomics), and anti-rabbit IgG (1:10000, Jackson). The ChIP assay was performed using the Pierce Agarose ChIP kit (Pierce; Thermo Scientific, Rockford, IL, USA) according to the manufacturer's protocol. The antibody used in ChIP analysis was p53 (2527; 1:200; CST). To amplify the potential p53-binding sites, specific primers for PCR were listed in supplementary table 2. RNA-ChIP was performed as described previously [23, 45, 46]. Immunoprecipitation for RNA-ChIP was performed using the following antibodies: p68 (05-850; Upstate) and Drosha (ab12286; Abcam). Specific primers for RNA-ChIP had the following sequences: forward primer (5′-GCCCAATGGCATAGCAAGA-3′) and reverse primer (5′-GGCCAGTCATGCTTACAGTCAC-3′).

### Cell proliferation, migration and invasion assays

Cell proliferation assays were performed as described previously [44]. Transwell invasion assays were performed according to the manufacturer's protocol (BD Biosciences, San Jose, CA, USA). Briefly, cells (1×10^5^) transfected with different small nucleotides for 48 h were added to the upper chamber of transwell filters coated with matrigel (or none) in serum-free medium. The cells that invaded the lower chamber were stained with Crystal violet stain and counted after 24 h of incubation at 37°C with 5% CO2. Photographs were taken at 24 h postmigration or postinvasion (magnification, 400×). Each experimental group had two replicates, and three fields in each replicate were randomly chosen for quantification of invasive cells.

### Co-immunoprecipitation

Co-immunoprecipitation was performed as described previously [21]. The following antibodies were used: anti-p53 (9282; 1:200; CST), anti-p68 (ab128928; 1:100; Abcam) and anti-Drosha (ab12286; 1:100, Abcam).

### Immunofluorescence

Briefly, HEC-1B cells were seeded on the confocal laser dish at a density of 5 × 10^4^ cells/ml and cultured for 24 h. The cultured cells were washed 3 times with PBS and fixed with 4% paraformaldehyde (Sigma-Aldrich, St. Louis, USA) for 30 min. After blocking, the cells were incubated first with anti-E-cadherin (3195; 1:100; CST), anti-N-cadherin(13116; 1:200; CST), anti-Vimentin (5741; 1:200; CST) or anti-Snail (ab180714; 1:50; abcam) antibody overnight at 4°C, and then with Cy3-conjugated or FITC-conjugated goat anti-rabbit IgG antibody (1:200; Abcam, Cambridge, UK) and 5 mg/ml DAPI (Sigma-Aldrich) at room temperature for 30 min. Then, the cells were observed under a laser scanning confocal microscope (Leica, Heidelberg, Germany) with emission wavelengths of 518 nm, 570 nm, and 461 nm.

### Construction of reporter plasmids and luciferase assay

The wild-type 3′UTR of *EZH2* was amplified using forward primer(5′-ATAGGCCGGCATAGACGCGTCATCTGCTACCTCCTCCCC-3′, MluI) and reverse primer(5′-AAAGATCCTTTATTAAGCTTGATTCAACAAGGACAAGTTCAAGTATTCTTTATTC-3′, hindlII), and the counterpart mutant 3′UTR was amplified using forward primer(5′-ATAGGCCGGCATAGACGCGTCATCTGCTACCTCCTCCCC-3′, MluI) and reverse primer(5′-AAAGATCCTTTATTAAGCTTGATTCAACAAGGACAAGTCTTCCGGTTCTTTATTCAAAGTTGAAAAATG-3′, hindlII) (The underlined primer sequence indicates the binding sites for MluI and hindIII). Amplicons were sub-cloned into MluI/hindlII sites of the pMIR-REPORT luciferase vector (Ambion) downstream of the luciferase gene to produce pMIR-EZH2-3′UTR-WT and pMIR-EZH2-3′UTR-MT, respectively. The luciferase reporter assay was performed as previously described [44]. Correct insertion was confirmed by gel electrophoresis and DNA sequencing.

### Animal experiments

Animal experiments were approved by the Ethics Committee for Animal Experimentation of Shanghai Jiaotong University and performed as described previously [44].

### Statistical analysis

All statistical analysis were performed using SPSS 18.0 and GraphPad Prism. Bars show mean ± SD and differences were evaluated using one-way ANOVA for 3-group comparisons and *t*-tests for 2-group comparisons. The probability of *P* < 0.05 was considered to be statistically significant. All experiments were repeated three times independently.

## SUPPLEMENTARY MATERIAL FIGURES


