# Supplementary material for: Mutant p53 induces EZH2 expression and promotes epithelial–mesenchymal transition by disrupting p68-Drosha complex assembly and attenuating miR-26a processing
Source: Oncotarget. 2015 Nov 18;6(42):44660–74. doi: 10.18632/oncotarget.6350 (PMC4792583; doi:10.18632/oncotarget.6350)
Supplement: Supplementary file 1 [file oncotarget-06-44660-s001.pdf]

Mutant p53 induces EZH2 expression and promotes epithelial–mesenchymal transition by disrupting p68-Drosha complex assembly and attenuating miR-26a processing

Supplementary Material

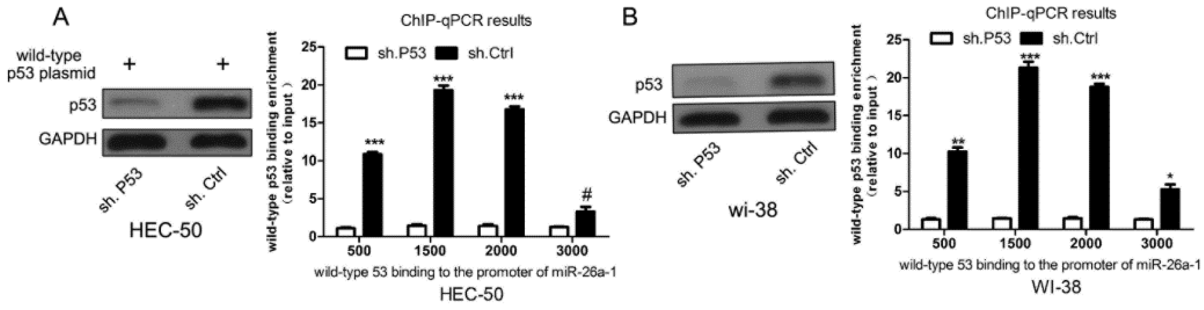

Supplementary figure 1. Analysis of WTp53 binding to the regulatory regions of miR-26a-1

A. A summary of ChIP assay results for WTp53 binding in HEC-50 cells transfected with sh.p53 and sh.Ctrl expression plasmid, respectively (***P＜0.001, #P=0.0509). **B.** A summary of ChIP assay results for WTp53 binding on 30th passage wi-38 cells transfected with sh.p53 and sh.Ctrl expression plasmid, respectively (***P＜0.001, ** P=0.002, *P=0.0138).

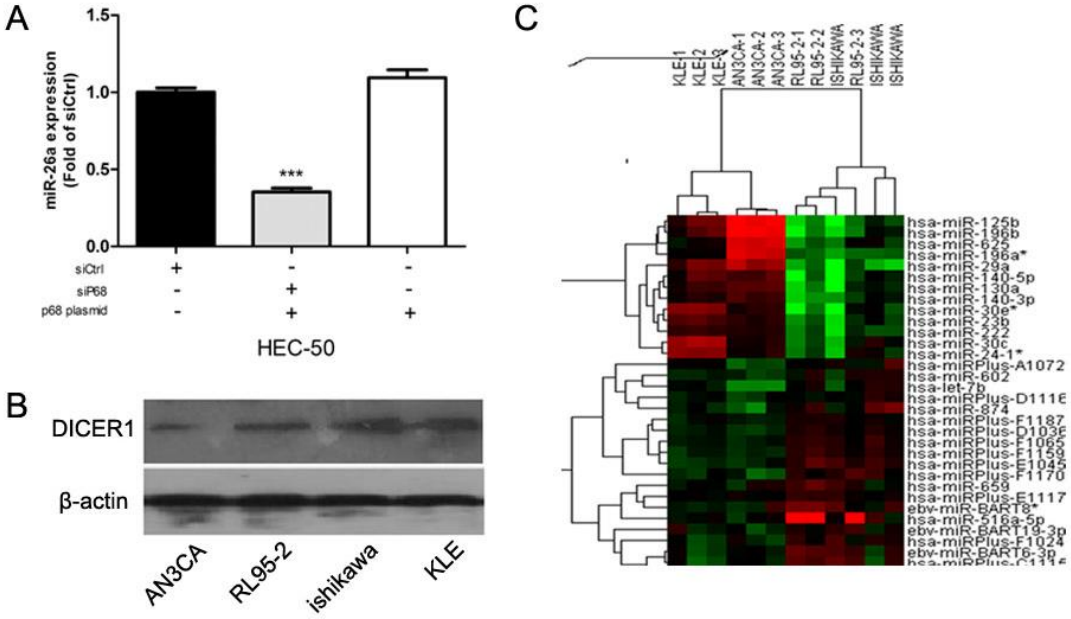

Supplementary figure 2. **DICER1 and miRNA expression in four EC cell lines**

A. MiR-26a expression decreased after p68 knockdown and was rescued by re-expression of p68 (***P=0.005). **B.** DICER1 protein was examined using western blot. **C.** MiRNA array showed that let-7b was low expression in AN3CA and KLE cells compared with Ishikawa and RL95-2. Red indicates high expression and green indicates low expression.

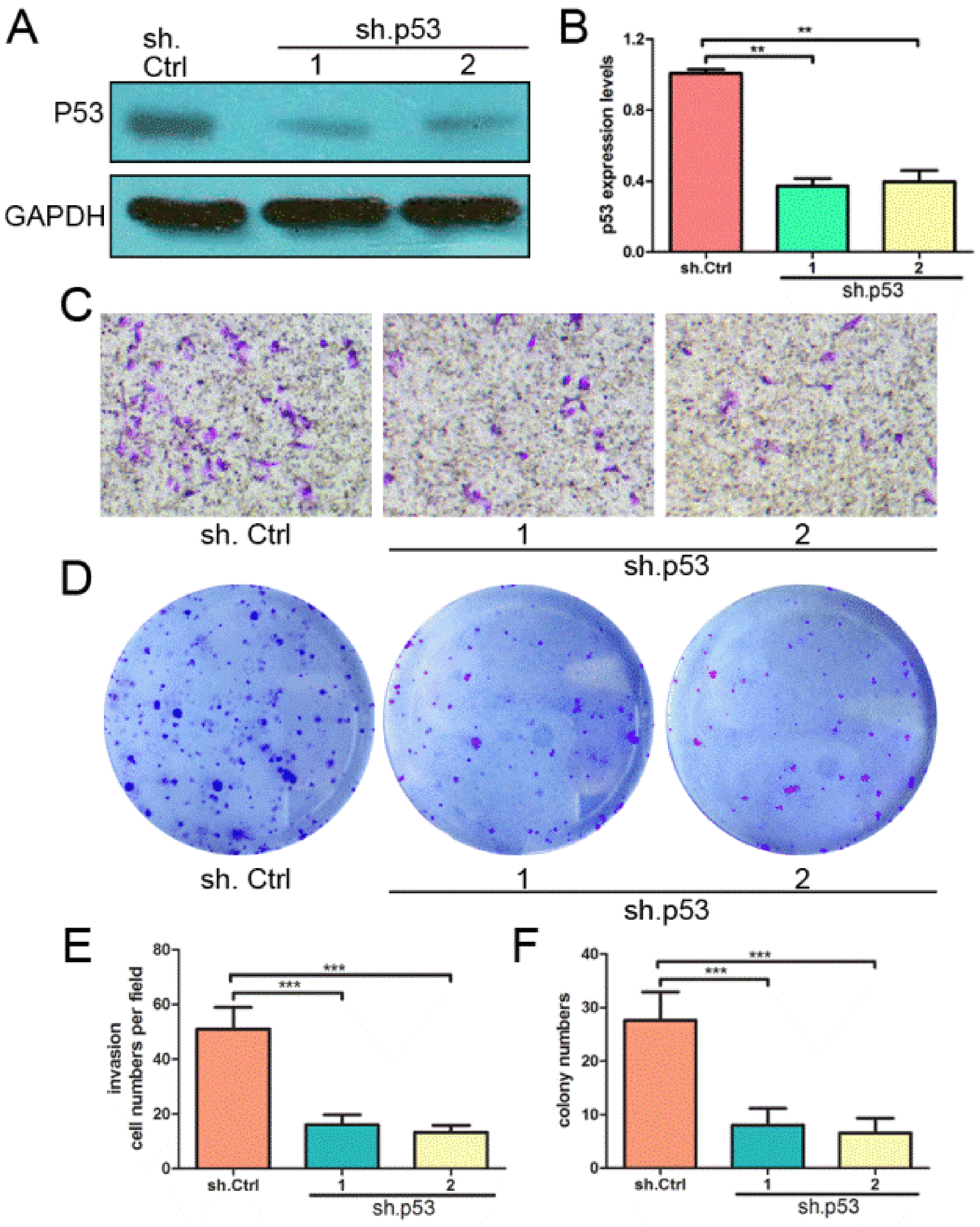

Supplementary figure 3. mutp53 knockdown impairs HEC-1B cell invasion and colony formation

A~B. Protein and mRNA of mutp53 were evaluated using western blot and qRT-PCR after stable knockdown mutp53. **C.** Representative transwell invasion images. **D.** Representative colony formation images. **E.** Quantification of C. **F.** Quantification of D.
